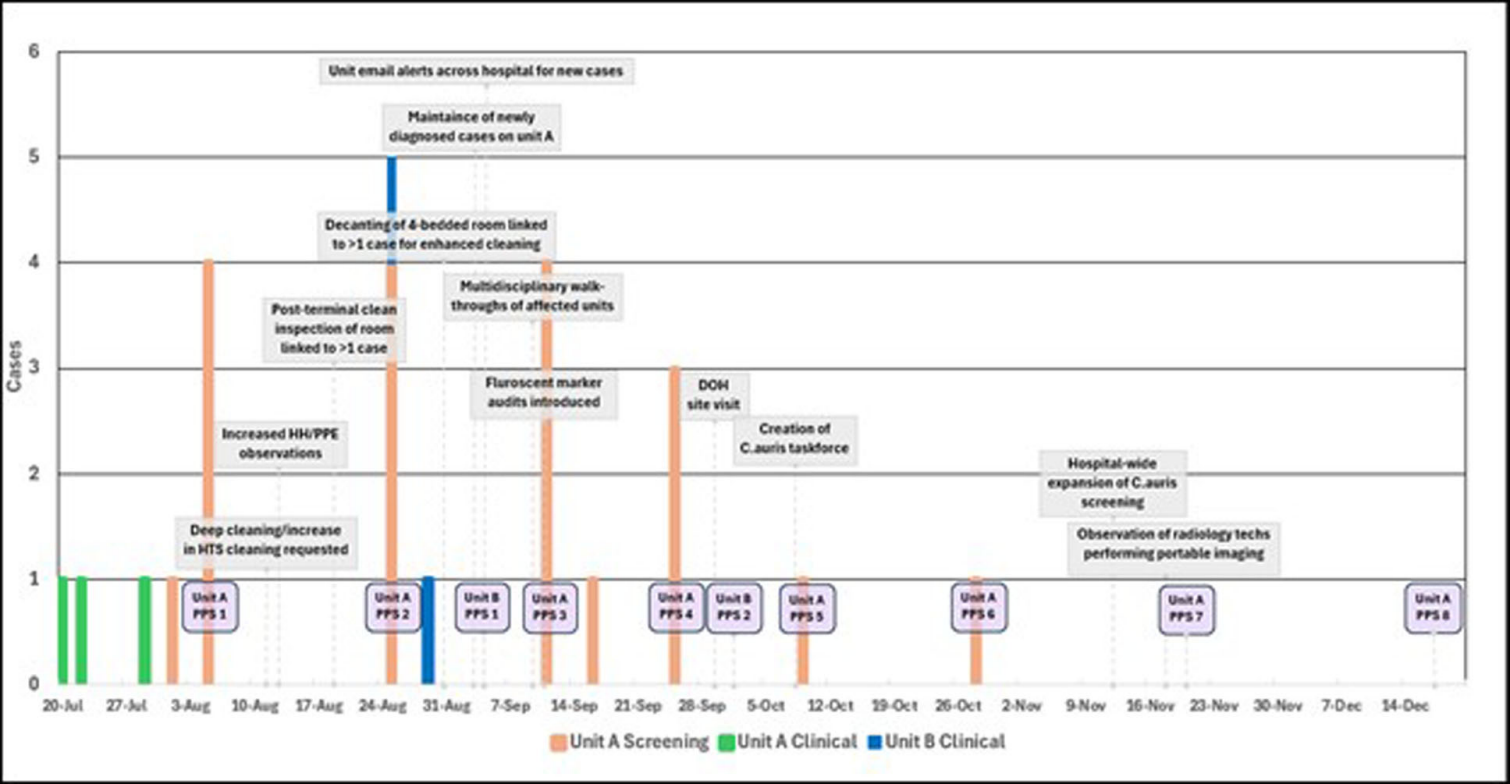# 238 A Structured, Multidisciplinary Approach to Reducing Colorectal Surgical Site Infections

**DOI:** 10.1017/ash.2026.10613

**Published:** 2026-06-23

**Authors:** Heidi Torres, Krystal Balzer, Christine Hatola, Allison Sladek, Kristen Magnuski, Robert Birkhead, Stephanie O’Neil, Cliff Dryden, Allen Uy, Lindsay Lief, Nekee Pandya, Lars Westblade, Lesley Covington, E. Yoko Furuya, Matthew Simon

**Affiliations:** 1 Weill Cornell Medicine; 2 New York Presbyterian Weill Cornell; 3 NewYork-Presbyterian/Weill Cornell Medical Center; 4 New York Presbyterian; 5 Weill Cornell Medical Center - New York Presbyterian Hospital; 6 New York Presbyterian Hospital; 7 Columbia University; 8 Weill Cornell Medical College

## Abstract

**Background:** Rates of Candida auris are rising across the US, particularly in New York. Due to C. auris’ affinity for environmental contamination and prolonged skin colonization, rapid implementation of comprehensive infection prevention & control (IP&C) measures is essential. Given the complexity and multidisciplinary involvement required for these efforts, we developed the C.A.R.E (Candida Auris Response & Engagement) framework in response to an inpatient outbreak to guide multidisciplinary efforts and provide a model for other facilities facing similar challenges with C. auris or other pathogens with extensive contact transmission. **Methods:** A C. auris cluster was identified in an 862-bed New York City academic medical center between July and October 2025. 23 cases were found involving a medical stepdown unit and adjacent intensive care unit. Baseline IP&C C. auris efforts include isolation precautions (gown and gloves), twice-daily room cleaning/disinfection, dedicated equipment, ATP testing of high touch surfaces (HTS) post terminal room cleaning, observations of personal protective equipment (PPE) use and hand hygiene, and screening of exposed roommates. After an August point prevalence surveillance (PPS) identified additional cases, we expanded interventions across various categories. The C.A.R.E. framework (Fig. 1) summarizes baseline and expanded efforts during this cluster. Figure 1. The C.A.R.E. (Candida Auris Response & Engagement) Framework to approaching a rise in C. auris cases. **Results:** Of 23 cases, 83% (19/23) were detected through surveillance. Clinical cases included bloodstream (3/4) and respiratory (1/4) sources. Shared rooms, either roommates or subsequent room occupants, were associated with 52% (12/23) of cases. Following implementation of the C.A.R.E. framework, further transmission ceased, confirmed by two negative rounds of PPS on each unit (Fig. 2). Figure 2. A timeline of C.auris cases identified on 2 units with corresponding IP&C effort implementation. To address identified gaps, a C. auris task force was created to improve IP&C processes. Key actions included clarifying equipment cleaning responsibility in collaboration with Environmental Services and Nursing, reevaluating the C. auris isolation category, and expanding screening for high-risk patients. Further, we incorporated fluorescent marker audits alongside ATP testing into routine practices to monitor environmental and equipment cleaning. While not all measures in the C.A.R.E. framework may be needed for every outbreak, facilities can adapt and scale interventions based on feasibility and outbreak severity. **Conclusions:** Effective prevention of C. auris transmission requires coordinated and multidisciplinary efforts. Establishing a structured response after early case detection can help close gaps in infection prevention that contribute to spread.

**Figure:**
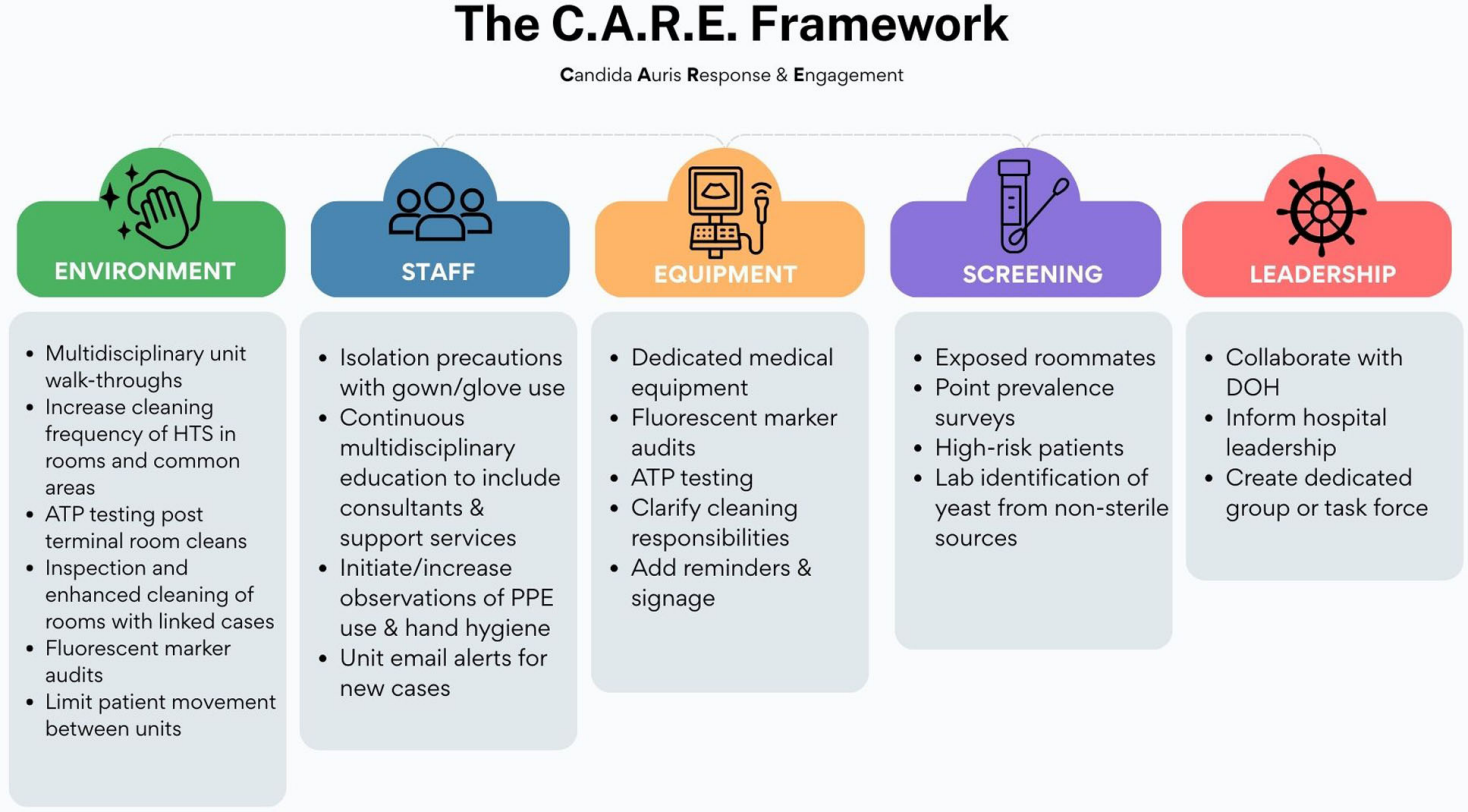

**Figure:**